# *Calicophoron daubneyi*—The Path Toward Understanding Its Pathogenicity and Host Interactions

**DOI:** 10.3389/fvets.2020.00606

**Published:** 2020-09-04

**Authors:** Erwan Atcheson, Philip J. Skuce, Nicola A. M. Oliver, Tom N. McNeilly, Mark W. Robinson

**Affiliations:** ^1^Microbes and Pathogen Biology, School of Biological Sciences, Queen's University Belfast, Belfast, United Kingdom; ^2^Disease Control, Moredun Research Institute, Pentlands Science Park, Edinburgh, United Kingdom

**Keywords:** *Calicophoron daubneyi*, rumen fluke, paramphistomosis, host-parasite interactions, pathogenicity, immunomodulation

## Paramphistomosis—An Established Infection in European Livestock

Infections by parasitic flukes are an important animal health and production concern for livestock producers worldwide. In the UK, and throughout the EU, liver fluke (*Fasciola hepatica*) historically has been a major focus for livestock farmers. However, in recent years, there has been a sharp increase in the incidence of rumen fluke (or paramphistome) infections in both sheep and cattle, such that they are now more common than liver fluke in many areas (1). The predominant rumen fluke species in the UK and Ireland has been confirmed as *Calicophoron daubneyi* (2), which appears to have spread from mainland Europe where it is common in countries such as France, Spain, and Belgium (1). Although there have been limited reports of *Paramphistomum leydeni* in Ireland (3, 4) and the Netherlands (5) the importance/pathogenicity of this species has not been investigated. Although the exact reasons for the increase in rumen fluke infections are not fully understood, the increase in warm wet summers and mild winters—conditions that favor *Galba truncatula*, the confirmed snail intermediate host of both *F. hepatica* and *C. daubneyi*—are thought to be the major contributing factor (6). It has also been suggested that *C. daubneyi* is adapting to out-compete the already endemic *F. hepatica* in their shared environment and snail intermediate host species (7). Furthermore, the widespread use of fasciolicides, to target liver fluke—most but not all are specific for liver fluke—may be giving *C. daubneyi* further competitive advantage over *F. hepatica*, such that *C. daubneyi* may eventually replace *F. hepatica* as the most prevalent endemic trematode in the UK/Ireland (8).

## *C. daubneyi*—A Growing Threat to UK Agriculture?

**Figure 1:**
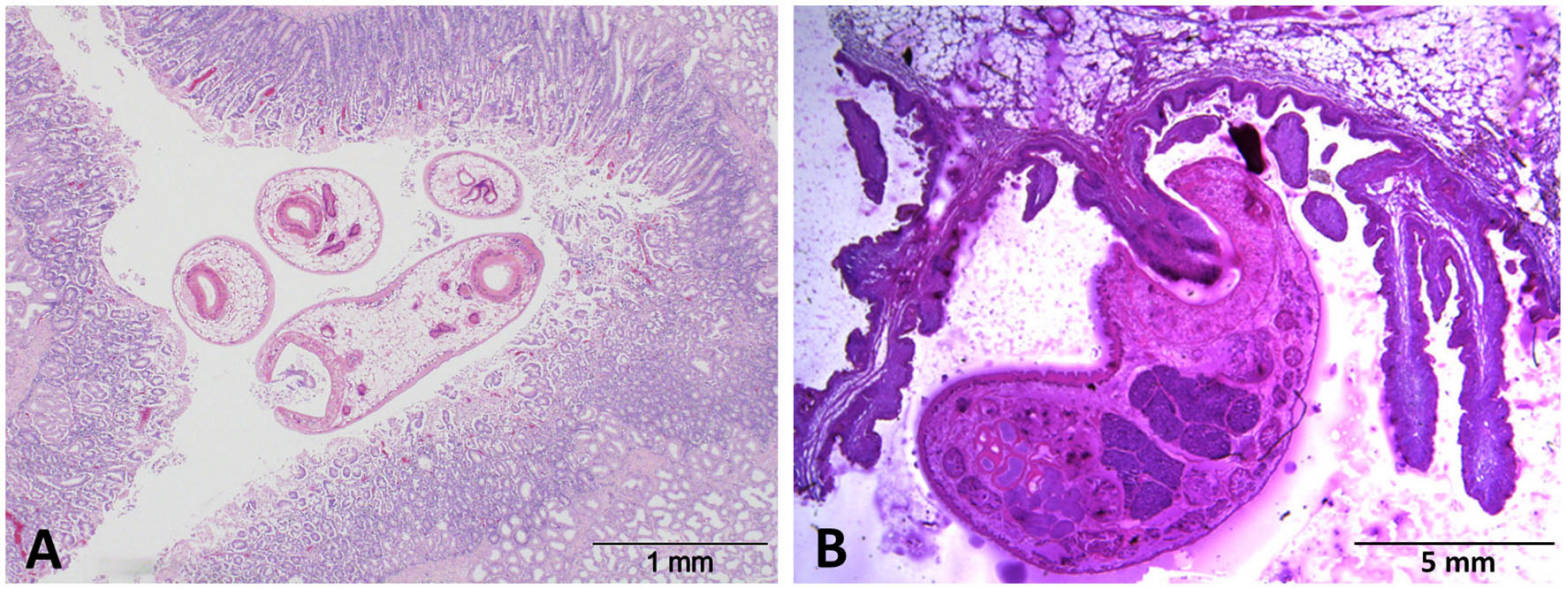
H&E-stained sections of **(A)** immature *C. daubneyi* larvae *in situ* within the duodenal mucosa and **(B)** an adult fluke attached to a rumen papilla *via* its muscular posterior acetabulum.

Acute, clinical paramphistomosis is caused when grazing livestock (typically youngstock in cattle or sheep of any age) ingest large numbers of rumen fluke metacercariae (cysts) from pasture, which then excyst *en masse* in the duodenum (Figure 1A). The newly excysted juvenile (NEJ) flukes then migrate into the intestinal submucosa causing significant damage to the host tissue (9). Large areas of damaged small intestine may hemorrhage, causing significant blood loss, and hypoalbuminaemia (10, 11). This initial damage, together with secondary bacterial infection, may trigger significant haemorrhagic enteritis, which often presents with fetid, black or bloody diarrhea (10, 12), and can result in mortality at this point. After feeding on the host tissue in the proximal small intestine (duodenum), immature paramphistomes migrate to the rumen where they mature (Figure 1B), with infections becoming patent at around 8–10 weeks post-infection (13). Although clinical disease is not currently associated with chronic paramphistome infections (which are common in the UK/Ireland, especially in cattle), post-mortem observations have noted both rumenitis and abomasitis in infected animals (14, 15), along with atrophy of the rumen papillae (1, 15). These effects may explain the (anecdotal) evidence from farmers that infected animals do not thrive, but respond well to oxyclozanide, the recommended treatment for rumen fluke.

Whilst paramphistomes are common in the UK/Ireland, clinical disease (i.e., poor condition/emaciation, with or without death, associated with acute infection) as described by Millar et al. (12) and Mason et al. (15) is still relatively rare. However, fatal disease outbreaks, linked to significant immature parasite burdens, are on the rise in both sheep and cattle (8, 11, 12, 15). For example, in a particularly severe outbreak on an Irish farm, 6-months old dairy heifers ingested an estimated 5,334 metacercariae each day over 3 weeks (8). Indeed, the Disease Surveillance and Investigation Branch of the Agri-food and Biosciences Institute (Northern Ireland) are seeing an increasing number of sudden deaths that showed no clinical presentation other than acute rumen fluke infection at post-mortem examination—particularly during years with mild wet conditions during the spring and early summer (Prof. Bob Hanna, personal communication, 2019). With the increasing prevalence of rumen fluke in the UK/Ireland, outbreaks of clinical (and fatal) paramphistomosis may rise further in the future.

## With Limited Treatment Options, Sustainable Control of Rumen Fluke Infection Looks Challenging

Given the rapid clinical progression associated with acute paramphistomosis, swift diagnosis, and timely interventions will be key in the treatment of disease outbreaks. However, neither of these is straightforward. ELISA-based diagnostic tests have yet to be developed for rumen fluke infections, [the current *F. hepatica* coproantigen test does not cross-react with *C. daubneyi*; Kajugu et al. (16)] leaving fecal egg counts or post mortem as the only options to identify chronic or acute disease, respectively (1). In addition, whilst some compounds show efficacy against the infection, there are currently no licensed anthelmintics available to control rumen fluke in the UK or Ireland. Closantel showed high activity in cattle when given as an oral drench (17) but had limited/no effect when injected subcutaneously (18, 19). In contrast, oxyclozanide has consistently shown high efficacy against juvenile and adult rumen fluke in cattle and goats (18, 20). However, reliance on a single drug for treatment of this potentially fatal parasite infection is not sustainable, and particularly worrying, since the emergence of drug-resistant rumen fluke populations following oxyclozanide mono-therapy would be inevitable. This situation will leave animal producers with little to combat the disease (agronomic approaches to control rumen fluke would likely be as effective as those for liver fluke since they use the same snail intermediate host). Consequently, a vaccine that would prevent rumen fluke infection and/or limit damaging intestinal pathology would be beneficial.

## Rumen Fluke May Be More Amenable To Control by Vaccination Than Liver Fluke

Field observations indicate that previous exposure of livestock to rumen fluke provides protection against massive infections that would typically cause acute paramphistomosis (9). For example, during a fatal outbreak of paramphistomosis caused by *Paramphistomum ichikawai* in lambs, adult ewes exposed to the same infected pasture harbored adult rumen flukes but had few immature intestinal stage parasites and were unaffected by the disease (21). These findings are supported by experimental studies in which sheep, cattle, and goats were successfully immunized with *Paramphistomum microbothrium* metacercariae, routinely reaching >99% protection against challenge infection (22, 23). Interestingly, these studies showed that immunization with adult worms (delivered orally to establish infection directly in the rumen) was not effective and that protective immunity requires exposure of the duodenum to NEJ-derived antigen. In contrast, host rumen fluke burdens have been shown to grow with age (24) indicating that protective immunity may not be widespread under field conditions. Nevertheless, there is more evidence for immunity to re-infection compared to liver fluke where natural acquired immunity is limited in cattle and less so in sheep (25). As a result, attempts to vaccinate livestock against liver fluke have been largely unsuccessful, despite considerable research effort (26).

## Research Priorities for a Better Understanding of Rumen Fluke Pathogenicity

Despite their prevalence, our knowledge of many aspects of basic rumen fluke biology, and how they interact with their hosts, is limited. This is in contrast to *F. hepatica*, the other major endemic trematode species in the UK, for which we have a very good understanding of the key metabolic, biochemical and molecular mechanisms underpinning invasion, virulence, and development, as well as a clear picture of the immune responses (in rodent and ruminant hosts) which define the cellular and molecular signatures of liver fluke infection (27–31).

Here, we list some research priorities, for *C. daubneyi*, based on the need to better understand the pathogenicity of this species and how the ruminant host responds to infection.

### Sources of Viable *C. daubneyi* Metacercariae

Currently, the only source of *C. daubneyi* metacercariae in the UK is Ridgeway Research Ltd. (St. Briavels, UK). Whilst it is possible to obtain large numbers of metacercariae by washing them directly from significantly contaminated pasture (8), access is seasonal and there is always a danger that these washings will contain cysts of *F. hepatica* or other trematode species. Clearly, establishment of other sources of experimentally-produced rumen fluke metacercariae would be beneficial to the research community.

### Establish *in vivo* Models of Infection

At present, *in vivo* models of *C. daubneyi* infection (e.g., using cattle or sheep) are lacking. Establishment of such models, using defined doses of metacercariae, would allow basic parasitological observations to be made, such as the timing of fluke development/patency (currently unknown) or the quantification of egg output. Moreover, *in vivo* models of *C. daubneyi* infection would allow correlates of fluke burden with egg output to be determined which would validate copromicroscopic techniques for detection of chronic paramphistomosis. The timing and extent of host pathology associated with acute rumen fluke infection could also be more accurately assessed and how this correlates with the burden of immature larvae in the duodenum. Characterization of the host immune response to rumen fluke infection, and how this changes as infection progresses to chronicity, would be of particular value and would facilitate vaccine trials and the development of immune-based diagnostic tools. Since a rumen fluke vaccine is likely to be some way off, repurposing of existing anthelmintics may be an attractive alternative and determining their efficacy *in vivo* would expedite this process.

### Develop *in vitro* Models of Acute Infection

We recently developed a new technique for excystment of *C. daubneyi* metacercariae (32). This consistently gave >80% excystment and has allowed us to study the infective NEJ stage *in vitro*. This development could permit detailed investigations into *C. daubneyi* infectivity, if suitable *in vitro* infection models can be developed. These could be based upon gut loop assays which have been used to probe mechanisms of secretion in *Fasciola gigantica* NEJs (33) and to determine the role of cathepsins B and L in gut penetration by *F. hepatica* NEJs (34).

### Identify Key Virulence and Invasion Factors Secreted by *C. daubneyi*

We have recently shown (using transcriptome and proteome analysis) that, following excystment, *F. hepatica* NEJs rapidly undergo significant developmental changes, and secrete a specific battery of virulence-associated proteins in order to establish infection in the mammalian host (30). At present, only the secretome of adult rumen fluke has been characterized (32). Further work to investigate the tissue-invasion strategy, and virulence factors, used by *C. daubneyi* NEJs to establish new infections in the host duodenal submucosa are thus required. This may allow the development of vaccines or other treatments aimed at limiting the damaging pathology that is the hallmark of acute paramphistomosis.

### Characterize the Host Immune Response to *C. daubneyi* Infection

Helminths have the capacity to suppress or regulate both innate and adaptive immune responses to promote their survival within the host. Whilst the immunological parameters of infection are well-defined for nematodes and liver fluke (35–37) we know little about this during *C. daubneyi* infections. However, it is evident that, unlike liver fluke, there is considerable host immunity to re-infection with rumen fluke if sheep are re-challenged following priming with the intestinal phase of the primary infection (23). Studies, using experimentally infected sheep/cattle, would allow the pathological and immunological responses to both primary and secondary *C. daubneyi* infections to be investigated in order to define immune signatures of protection. Furthermore, this would allow investigation of the immune outcomes of co-infection of rumen fluke with *F. hepatica* and/or gastrointestinal nematodes.

## Conclusions

With the possibility of clinical outbreaks of paramphistomosis increasing in the future, it is important that we begin to investigate the basic biology of *C. daubneyi* now, with emphasis on host pathology and immune evasion. Uniquely, studies from the 1960's indicate that protective immunity occurs for paramphistomosis and that successful vaccination could be possible. However, these observations require detailed follow-up investigation using the raft of immunological reagents, biological resources and molecular tools available today. Although some way off, a vaccine could prolong the useful life of oxyclozanide which is currently the only drug recommended, although not licensed, for rumen fluke infection. Pursuit of the basic research areas outlined here would be a starting point for such translational developments.

## Author Contributions

MR, TM, and PS conceived the research priorities described. EA, MR, NO, TM, and PS wrote the manuscript. NO contributed figures. All authors contributed to the article and approved the submitted version.

## Conflict of Interest

The authors declare that the research was conducted in the absence of any commercial or financial relationships that could be construed as a potential conflict of interest. The handling Editor declared a past co-authorship with one of the authors PS.
